# Supplementary material for: Computational investigation of O2 diffusion through an intra-molecular tunnel in AlkB; influence of polarization on O2 transport
Source: Chem Sci. 2017 Jul 5;8(9):6230–8. doi: 10.1039/c7sc00997f (PMC5628400; doi:10.1039/c7sc00997f)
Supplement: Supplementary file 5 [file SC-008-C7SC00997F-s005.pdf]

Electronic Supplementary Material (ESI) for Chemical Science.
This journal is © The Royal Society of Chemistry 2017

Computational Investigation of O_2 Diffusion Through an Intra–molecular Tunnel in AlkB; Influence of Polarization on O_2 Transport

Hedieh Torabifard* G. Andrés Cisneros†

July 6, 2017

Tunnel	Length (Å)	Bottle-neck Radius (Å)	Curvature	Priority-throughput
Blue	15.8	0.93	1.23	0.54
Red	16.4	0.93	1.47	0.49
Orange	18.1	0.93	1.30	0.40
Green	31.4	0.93	1.97	0.23

Table S1: Tunnel properties for crystal structure

Tunnel	Number of Occurrence	Average Length (Å)	Average Bottle-neck Radius (Å)	Average Curvature	Average Priority-throughput
Blue	77 (29.4%)	17.1	1.00	1.32	0.41
Red	74 (28.2%)	15.8	1.01	1.42	0.45
Other	111 (42.4%)	–	–	–	–
Total	262	–	–	–	–

Table S2: Number of occurrence and properties of each tunnel from 250 snapshots of MD simulations

*Department of Chemistry, Wayne State University, Detroit, MI 48202
†Department of Chemistry, University of North Texas, Denton, TX 76203, E-mail: andres@unt.edu

	$\Delta\Delta E_{Coul}$	$\Delta\Delta E_{vdW}$	$\Delta\Delta E_{Tot}$
WT	0	0	0
W178Y	-48.5	4.4	-44.1
W178A	-28.6	14.5	-14.1
W178P	6.7	8.3	15.0

Table S3: Difference between ΔE of mutants and WT. ΔE is the sum of all intermolecular interaction energies for all residues in each system (except water). Energies of Coulomb, van der Waals and total non-bonded interactions are given in kcal/mol.

H-donor	H-accepotor	Run 1	Run 2	Run 3	Run 4	Average
E136(OE1)	R183(HH12–NH1)	43.7	28.4	51.9	66.7	47.7
	R183(HH22–NH2)	45.4	58.0	30.7	64.7	49.7
	R210(HH21–NH2)	23.2	29.0	47.6	67.1	41.7
	R210(HE–NE)	40.0	50.7	35.1	21.3	36.8
E136(OE2)	R183(HH12–NH1)	45.1	65.4	40.9	23.2	43.7
	R183(HH22–NH2)	42.4	28.7	43.7	16.8	32.9
	R210(HH21–NH2)	45.7	66.4	38.7	22.9	43.4
	R210(HE–NE)	42.6	32.9	45.1	56.8	44.4

Table S4: Hydrogen bond analysis for residues at red tunnel bottle neck. Atoms are labeled using PDB nomenclature. Values are percentage of simulation time in which the hydrogen bond is observed, averaged over four simulations of 500 ns each.

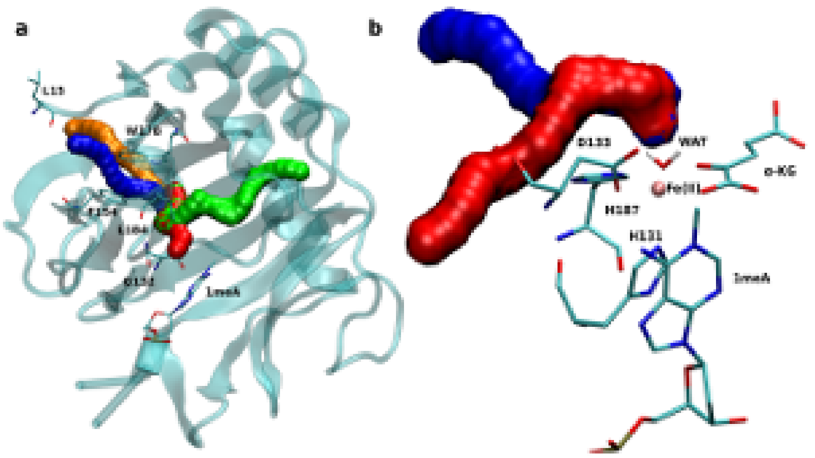

Figure S1: a) Tunnels obtained with CAVER for the crystal structure of AlkB. b) AlkB active site with blue and red tunnels. Water molecule which is at trans position to His131 can be replaced by O_2 molecule.

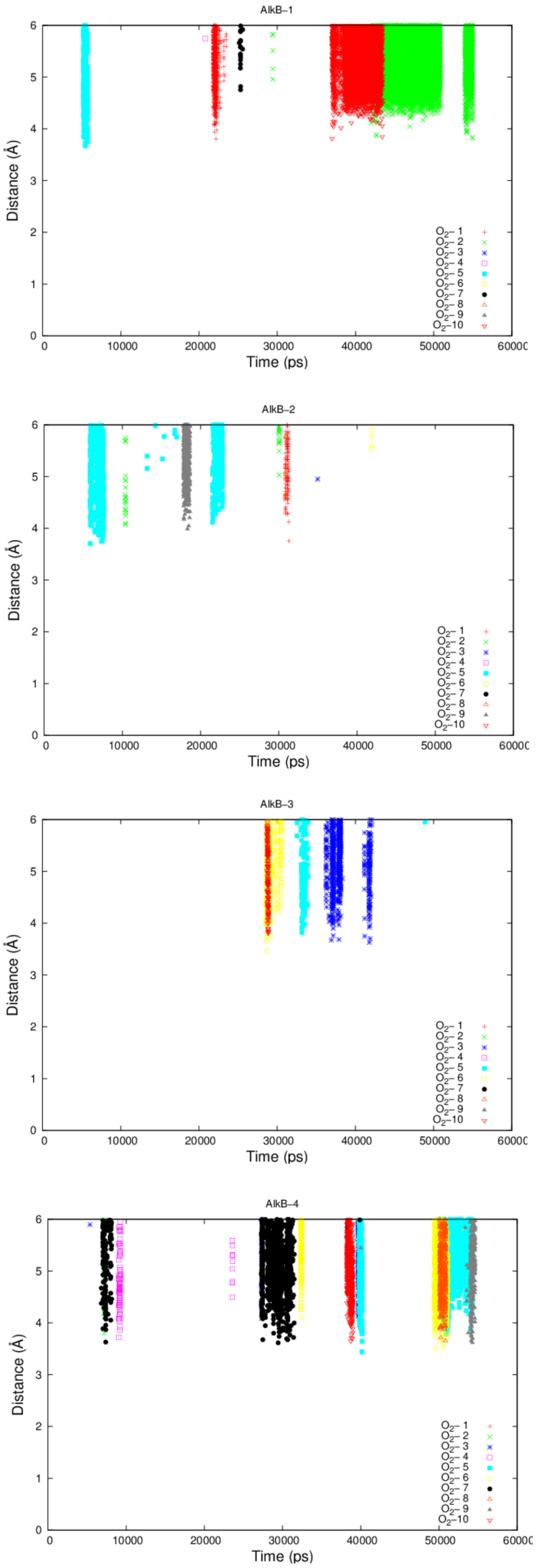

Figure S2: Distance between O_2 and Fe(II) in wild type AlkB for 4 independent simulations. The first one shows that 5 oxygen molecules diffuse into the tunnel. The second and third one show 4 oxygen molecules diffuse into the active site. Eight oxygen molecules diffuse into the active site in the fourth simulation. The distance less than 6 Å between oxygen molecules and iron considered as a complete entrance.

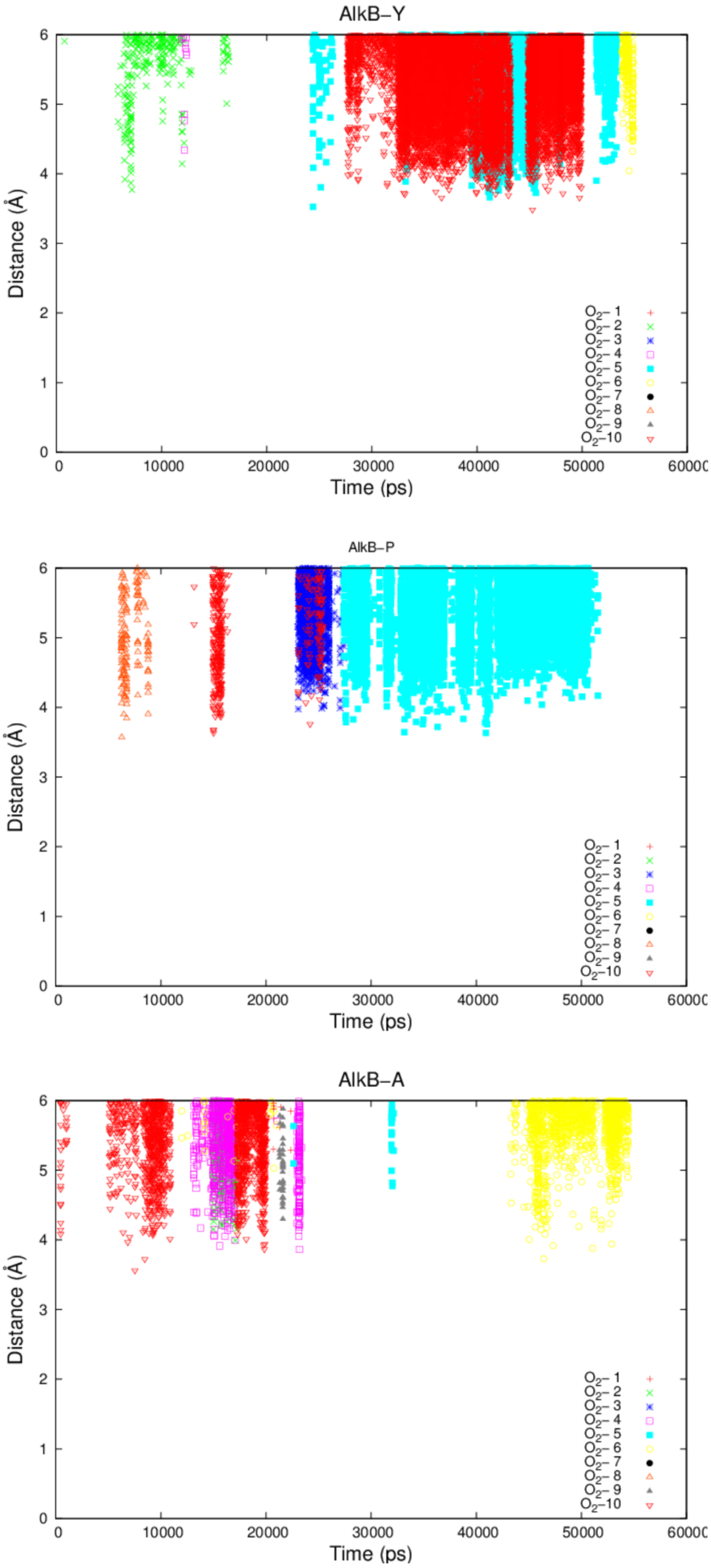

Figure S3: Distance between O_2 and Fe(II) for a) W178Y, b) W178P and c) W178A. 5 and 4 O_2 molecules diffuse into the active site in W178Y and W178P mutant, respectively. There are 6 O_2 molecules in W178A mutant active site. 4 O_2 molecules are delivered through blue tunnel and 2 O_2 molecules through a new pathway. On average 5 oxygen molecules diffuse into the active site. The distance less than 6 Å between oxygen molecules and iron considered as a complete entrance.

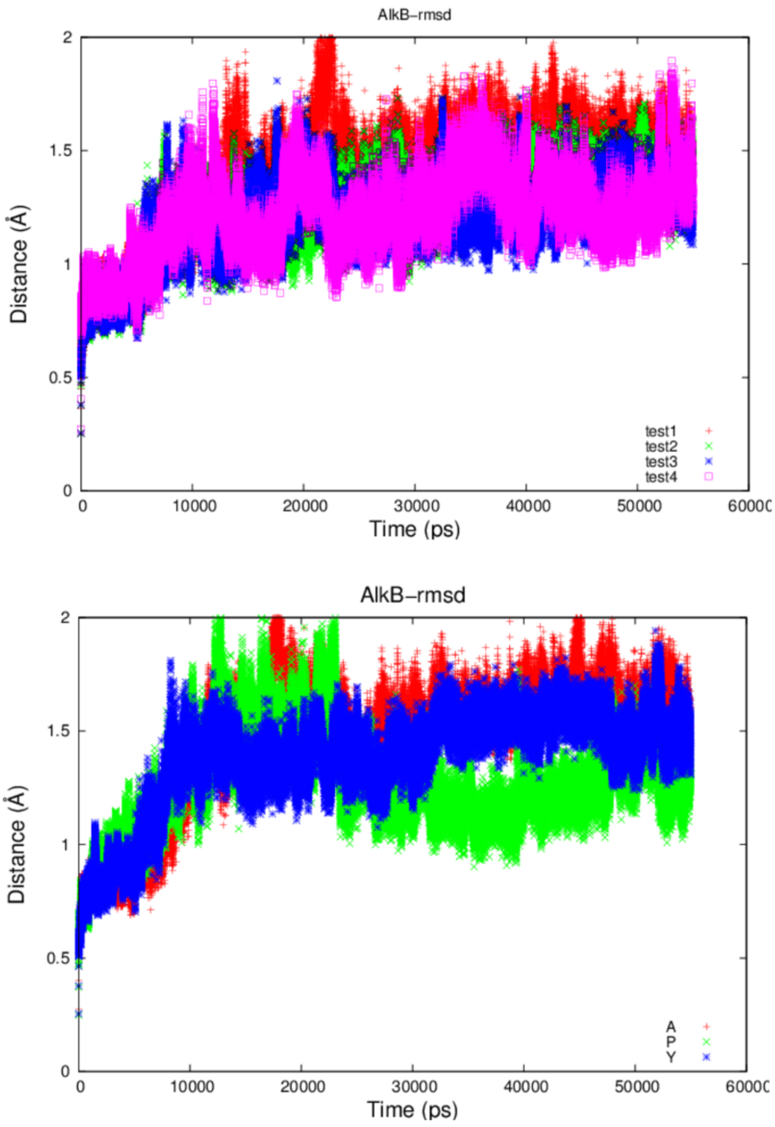

Figure S4: Protein backbone RMSD for a) WT AlkB b) W178A/P/Y mutants.

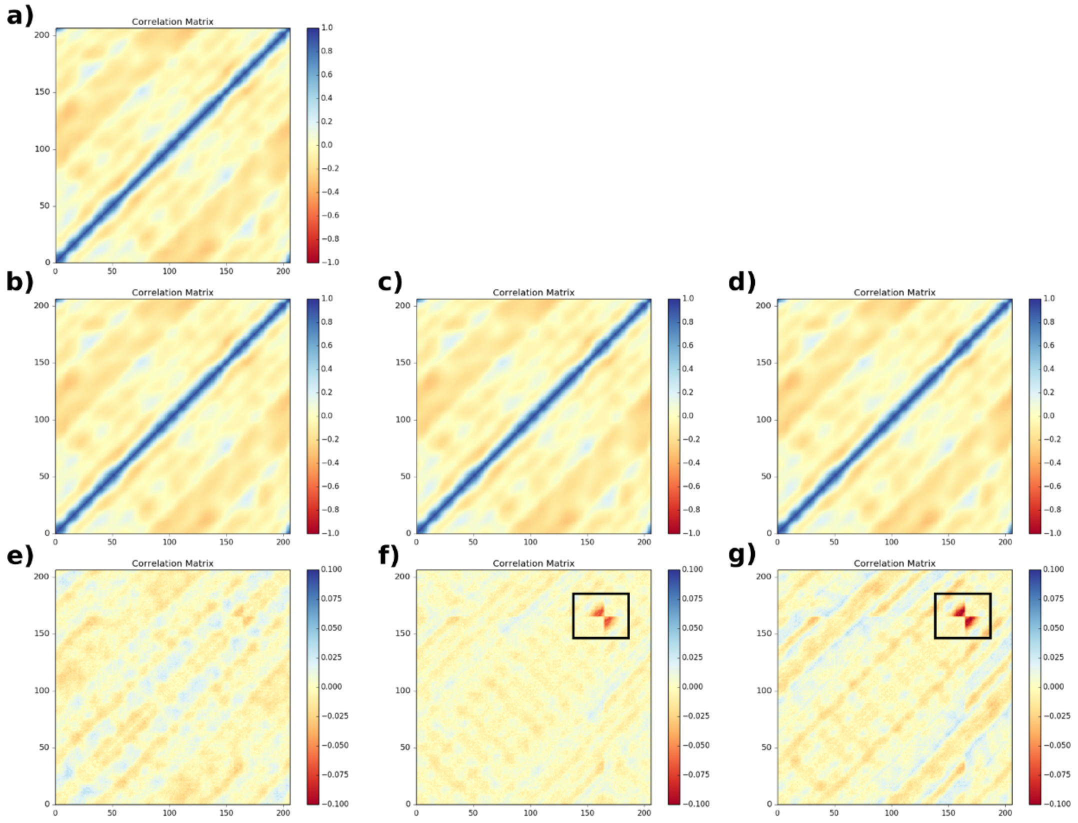

Figure S5: Protein motions may account for some differences in reactivity. The correlation plots for (a) WT, (b) W178Y, (c) W178P and (d) W178A. Correlation analysis by residue was carried out for WT, W178Y, W178P, and W178A mutants using the cpptraj module of Amber14, across the entire simulations. Residue pairs with correlated motions are shown in blue, while anti-correlated motions are shown in red. The correlation difference plots for (e) W178Y, (f) W178P and (g) W178A compare the mutant correlation plot to that of the WT and were calculated using an in-house python script. The range in plots was narrowed to -0.1 to 0.1 to highlight areas that appear different. Illustrative, regional changes in the single mutants are marked with boxes. For instance, residues 150–176 (corresponded to D163–I189) in W178P and W178A are anti-correlated. W178Y shows a pattern more consistent with the WT, suggesting that this mutant behave similar to WT.

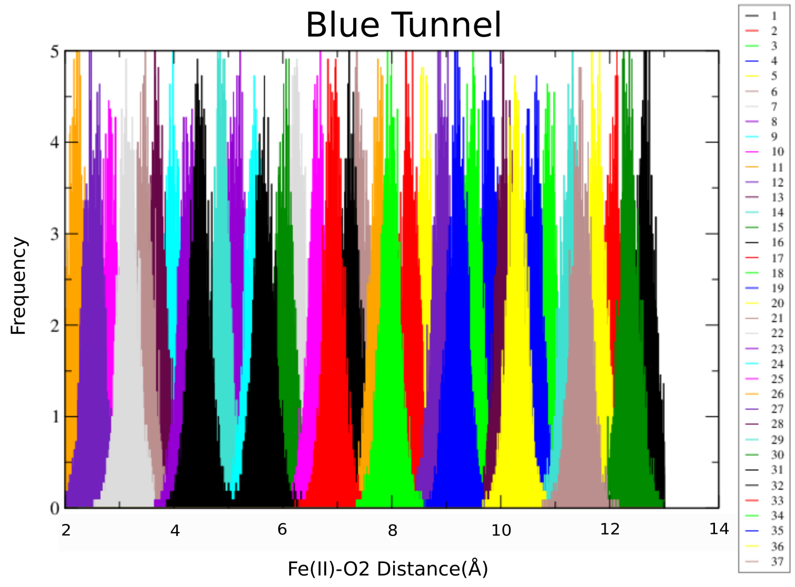

Figure S6: Histogram analysis for blue tunnel with AMBER force field. The coordinated water is included in this calculations.

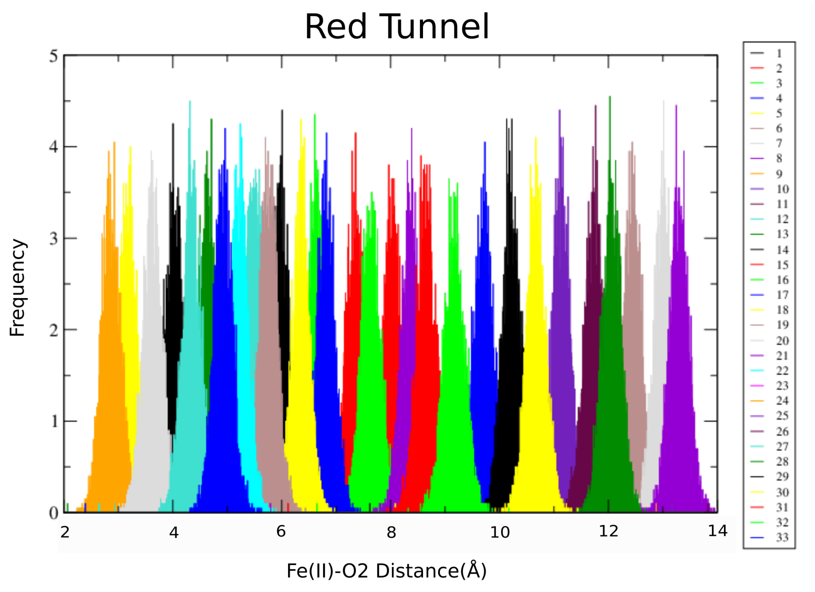

Figure S7: Histogram analysis for red tunnel with AMBER force field. The coordinated water is included in this calculations.

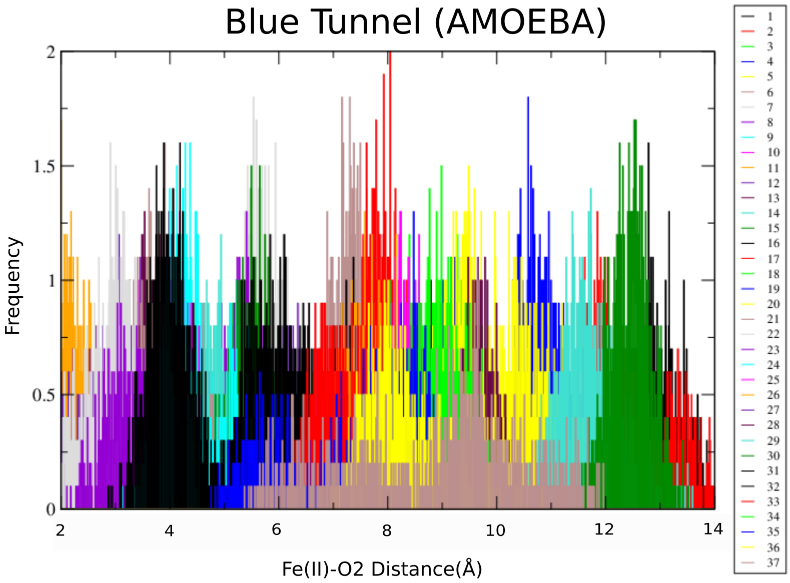

Figure S8: Histogram analysis for blue tunnel with AMOEBA force field. The coordinated water is included in this calculations.

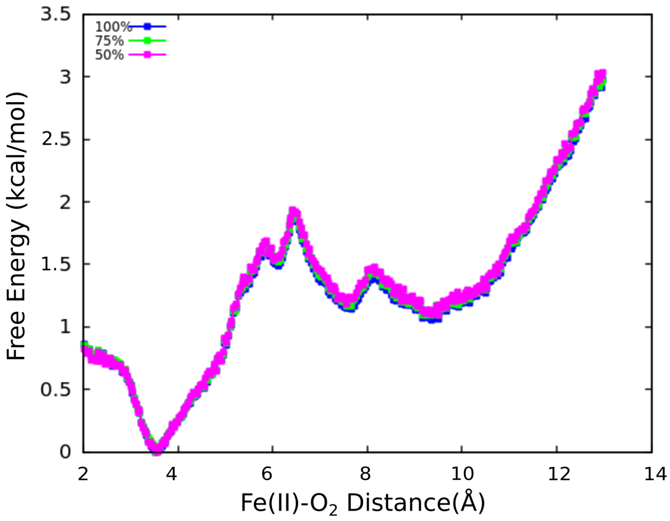

Figure S9: Bootstrapping analysis for blue tunnel with AMBER force field. The coordinated water is included in this calculations.

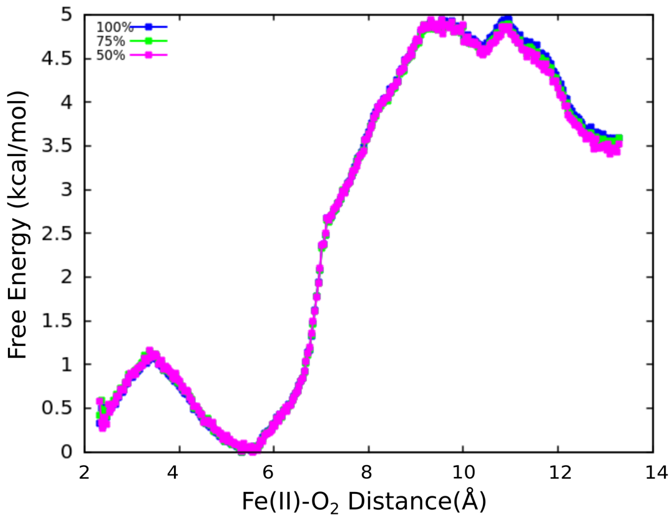

Figure S10: Bootstrapping analysis for red tunnel with AMBER force field. The coordinated water is included in this calculations.

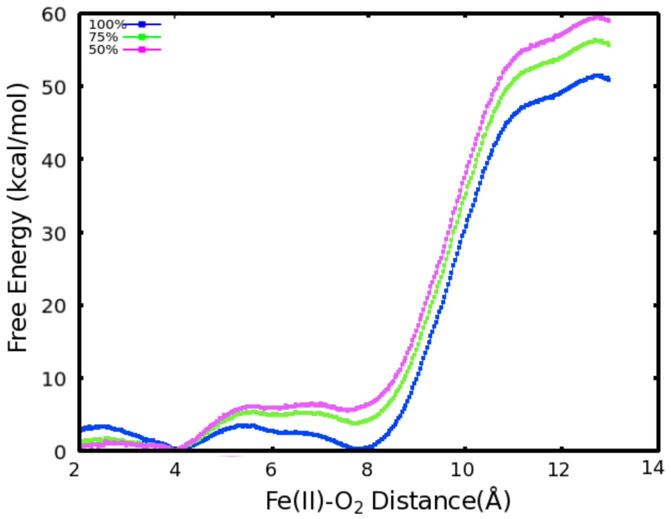

Figure S11: Bootstrapping analysis for blue tunnel with AMOEBA force field. The coordinated water is included in this calculations.

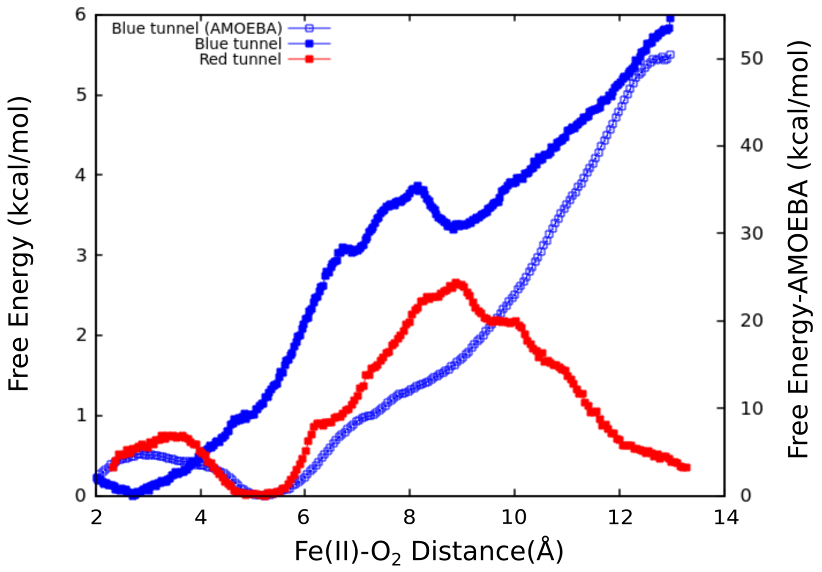

Figure S12: Calculated PMFs for intramolecular O_2 transport in AlkB. The PMF for the blue tunnel was calculated with the ff99SB, and AMOEBA force fields. For the PMF associated with transport along the red tunnel only the ff99SB potential was used. The coordinated water is deleted in this calculations.

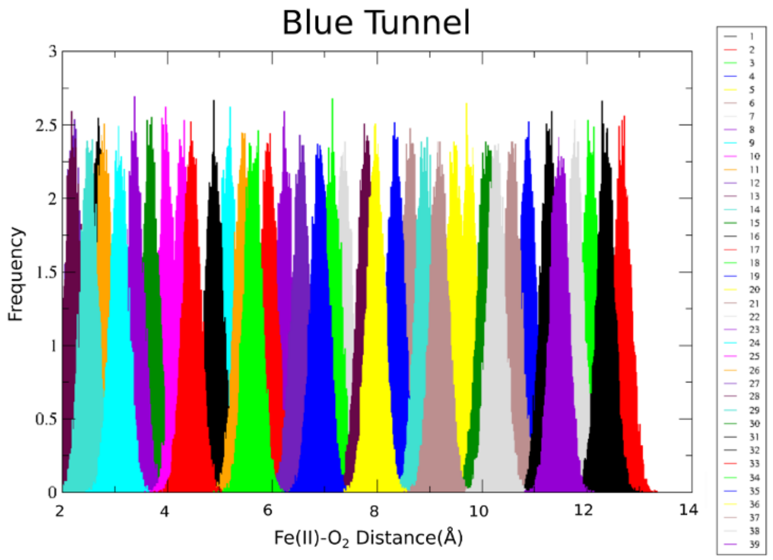

Figure S13: Histogram analysis for blue tunnel with AMBER force field. The coordinated water is deleted in this calculations.

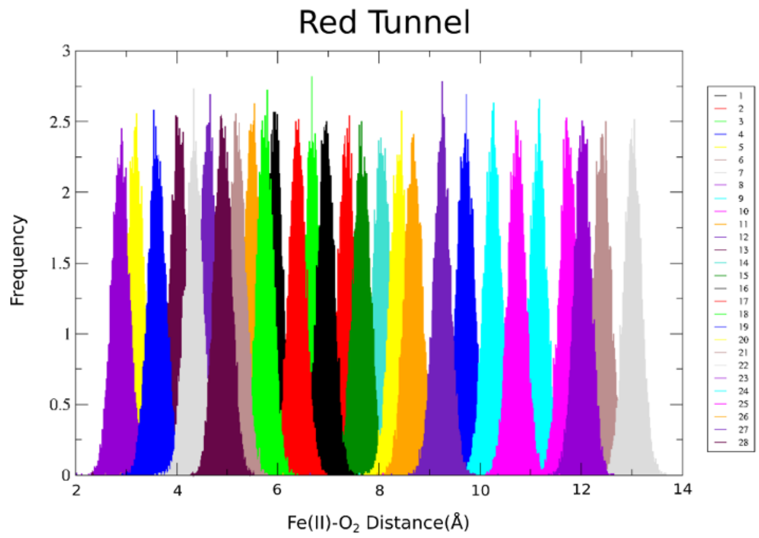

Figure S14: Histogram analysis for red tunnel with AMBER force field. The coordinated water is deleted in this calculations.

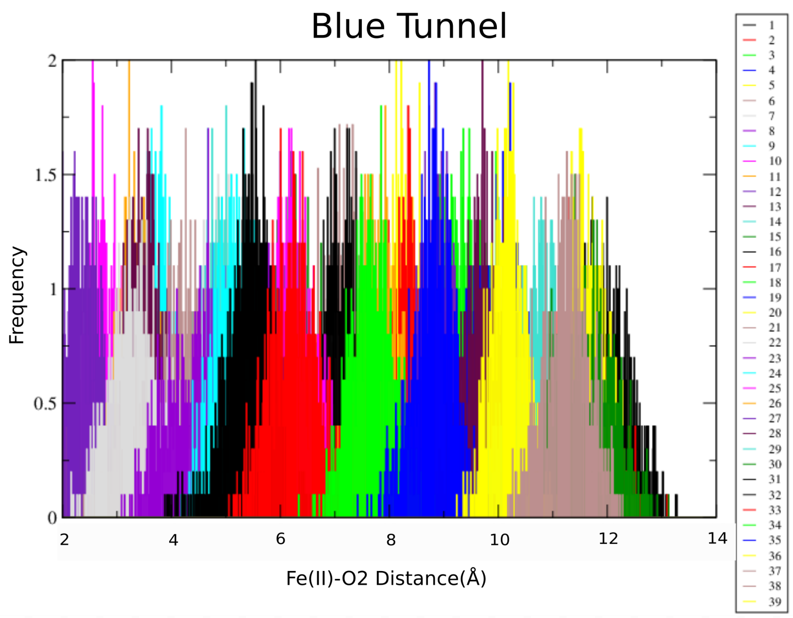

Figure S15: Histogram analysis for blue tunnel with AMOEBA force field. The coordinated water is deleted in this calculations.

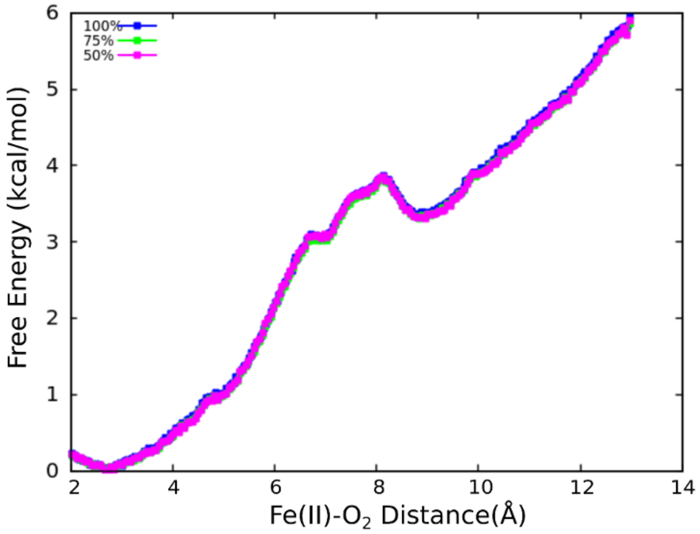

Figure S16: Bootstrapping analysis for blue tunnel with AMBER force field. The coordinated water is deleted in this calculations.

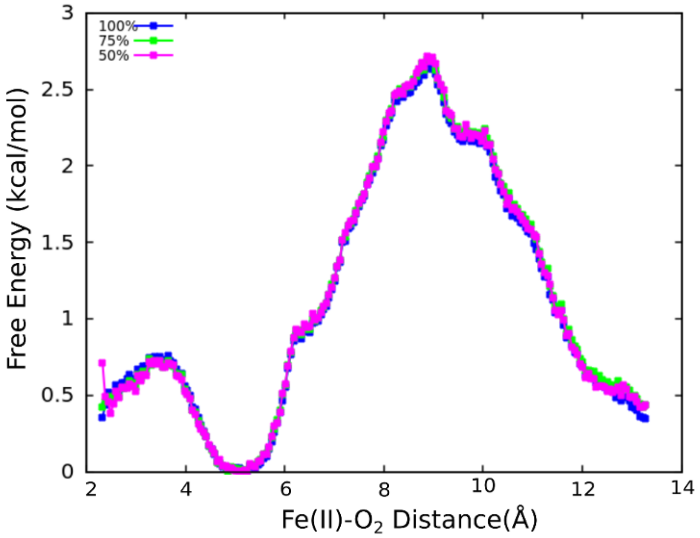

Figure S17: Bootstrapping analysis for red tunnel with AMBER force field. The coordinated water is deleted in this calculations.

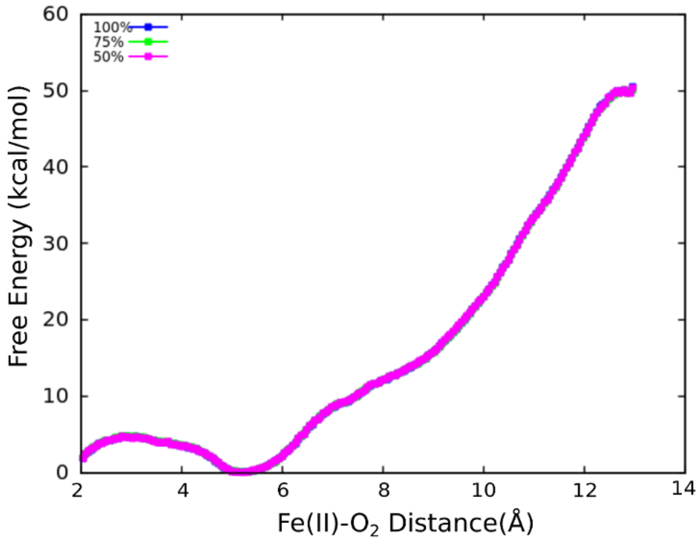

Figure S18: Bootstrapping analysis for blue tunnel with AMOEBA force field. The coordinated water is deleted in this calculations.
